# Lactate Regulates Rat Male Germ Cell Function through Reactive Oxygen Species

**DOI:** 10.1371/journal.pone.0088024

**Published:** 2014-01-31

**Authors:** María Noel Galardo, Mariana Regueira, María Fernanda Riera, Eliana Herminia Pellizzari, Selva Beatriz Cigorraga, Silvina Beatriz Meroni

**Affiliations:** Centro de Investigaciones Endocrinológicas “Dr. César Bergadá”, Consejo Nacional de Investigaciones Científicas y Técnicas (CONICET), Buenos Aires, Argentina; University Hospital of Münster, Germany

## Abstract

Besides giving structural support, Sertoli cells regulate the fate of germ cells by supplying a variety of factors. These factors include hormones, several pro- and anti-apoptotic agents and also energetic substrates. Lactate is one of the compounds produced by Sertoli cells, which is utilized as an energetic substrate by germ cells, particularly spermatocytes and spermatids. Beyond its function as an energy source, some studies have proposed a role of lactate in the regulation of gene expression not strictly related to the energetic state of the cells. The general hypothesis that motivated this investigation was that lactate affects male germ cell function, far beyond its well-known role as energetic substrate. To evaluate this hypothesis we investigated: 1) if lactate was able to regulate germ cell gene expression and if reactive oxygen species (ROS) participated in this regulation, 2) if different signal transduction pathways were modified by the production of ROS in response to lactate and 3) possible mechanisms that may be involved in lactate stimulation of ROS production. In order to achieve these goals, cultures of germ cells obtained from male 30-day old rats were exposed to 10 or 20 mM lactate. Increases in lactate dehydrogenase (LDH) C and monocarboxylate transporter (MCT)2 expression, in Akt and p38-MAPK phosphorylation levels and in ROS production were observed. These effects were impaired in the presence of a ROS scavenger. Lactate stimulated ROS production was also inhibited by a LDH inhibitor or a NAD(P)H oxidase (NOX) inhibitor. NOX4 expression was identified in male germ cells. The results obtained herein are consistent with a scenario where lactate, taken up by germ cells, becomes oxidized to pyruvate with the resultant increase in NADH, which is a substrate for NOX4. ROS, products of NOX4 activity, may act as second messengers regulating signal transduction pathways and gene expression.

## Introduction

Spermatogenesis is a long, complex and finely tuned process. Under physiological conditions, Sertoli cell/germ cell interactions play an important role in controlling the process of spermatogenesis. Besides giving structural support, Sertoli cells regulate the fate of germ cells by supplying a variety of factors. These factors include hormones, several pro- and anti-apoptotic agents and also energetic substrates. Lactate is one of the compounds produced by Sertoli cells, which is utilized as an energetic substrate by germ cells, particularly spermatocytes and spermatids [1], [2].

Beyond its function as an energy source, some studies have proposed a role of lactate in the regulation of gene expression not strictly related to the energetic state of the cells. In this context, Hashimoto et al. [3], working on the muscle cell line L6, have observed that lactate up-regulates genes related to its own metabolism by a mechanism that involves reactive oxygen species (ROS) production. It has to be born in mind that once lactate is taken up by the cells via the monocarboxylate transporters (MCTs), its conversion to pyruvate by the enzyme lactate deshydrogenase (LDH) is accompanied by NADH production, thus modifying the redox status of the cells, which might lead to a modification in the levels of ROS [4], [5]. It is well known that an excessive ROS production is harmful to the cell and in fact, it has been considered a cause of several pathological conditions. However, recent findings suggest that low and regulated ROS production may be relevant to cellular activity under physiological conditions [6]. Noteworthy, the initiation and/or proper functioning of several signal transduction pathways such as PI3K/Akt, p38-MAPK and Erk1/2 may be involved in the mechanism of action of ROS —now acting as signalling molecules [7], [8], [9].

So far, several reports have associated lactate provision with male germ cell metabolic needs [1], [2], [10], [11], [12]. However, no data are available on possible effects of lactate in ROS generation and in the regulation of other physiological aspects of these cells.

The general hypothesis that motivated this investigation was that lactate affects male germ cell function, far beyond its well-known role as energetic substrate. To evaluate this hypothesis we investigated: 1) if lactate was able to regulate germ cell gene expression and if ROS participated in this regulation, 2) if different signal transduction pathways were modified by the production of ROS in response to lactate and 3) possible mechanisms that may be involved in lactate stimulation of ROS production.

The results obtained herein are consistent with a scenario where lactate, taken up by germ cells, becomes oxidized to pyruvate with the resultant increase in NADH, which is a substrate for NOX4. ROS, products of NOX4 activity, may act as second messengers regulating signal transduction pathways –Akt and p38-MAPK- and gene expression –MCT2 and LDH C.

## Materials and Methods

### Materials

Tissue culture media Minimum Essential Media without pyruvate and L-alanine were purchased form GIBCO BRL (Life Technologies Ltd, Rockville, MD, USA). All other reagents were purchased from Sigma-Aldrich (St Louis, MO, USA).

### Ethics statement

Thirty-day-old Sprague–Dawley rats were obtained from an animal care unit (Animal Care Laboratory, Instituto de Biología y Medicina Experimental, Buenos Aires, Argentina). Animals were killed by CO_2_ asphyxiation according to protocols for animal laboratory use following the principles and procedures outlined in the National Institute of Health Guide for Care and Use of Laboratory Animals. The protocol was approved by the Ethical Commitee from the Instituto de Biología y Medicina Experimental (Ref.: CE 031/2013, IByME).

### Germ cell isolation and culture

Germ cells were isolated as previously described [13]. Testes were decapsulated and digested with 0.1% collagenase (C0130 Sigma-Aldrich) and 0.006% soybean trypsin inhibitor (T9003 Sigma-Aldrich) in Hanks' balanced salt solution (HBSS) for 5 minutes at room temperature. The collagenase solution was diluted 4-fold with HBBS and seminiferous tubules allowed to sediment for 2 minutes. The supernatant was discarded and the tubular pellet was washed twice with gentle shaking. Seminiferous tubules were cut into 2 mm segments and then digested with 0.05% collagenase, 0.003% soybean trypsin inhibitor and 0.003% deoxyribonuclease (DN25, Sigma-Aldrich) for 15 minutes at room temperature, while carefully transferring the suspension from one tube to another with a pipette. The suspension was diluted with one volume HBSS and material allowed to sediment for 5 minutes. The supernatant was transferred to a tube containing sufficient 2% bovine serum albumin (BSA) to make the final concentration 0.2% BSA. The suspension was allowed to settle for 10 minutes. Germ cells remaining in suspension were collected by centrifugation at 400×g for 3 minutes at 4°C. The resulting pellet was washed twice with HBSS containing 0.2% BSA and 0.003% deoxyribonuclease. The final cell pellet was resuspended in a 1∶1 mixture of Dulbecco's Modified Eagle's Medium-Ham's F-12 Medium with the addition of 15 mM NaHCO_3_, 100 IU/ml penicillin, 2.5 mg/ml amphotericin B, 20 mM Hepes, pH 7.4 (DMEM-F12) and seeded on a discontinuous four-layer (20%, 25%, 32%, 37%) Percoll density gradient. The gradient was centrifuged at 800×g for 30 minutes at 4°C. The fractions at the 25%–32% interface was collected. To remove Percoll, 4 volumes of DMEM-F12 were added and centrifugation at 400×g for 5 minutes at 4°C performed. Germ cells were resuspended in DMEM-F12 supplemented with 10 µg/ml transferrin, 5 µg/ml insulin, 5 µg/ml vitamin E and 4 ng/ml hydrocortisone. Germ cell preparations were seeded at a density of 2×10^6^ cell/ml in tissue culture flasks and cultured at 34°C in a mixture of 5% CO_2_∶95% air for 18 hours. During this initial period, the few Sertoli cells contaminating the germ cell preparation attached to the plastic surface. Purified germ cells were obtained by carefully removing the medium and centrifuging at 400×g for 5 minutes at 4°C, and resuspended in Minimum Essential Media without pyruvate and L-alanine (MEM) supplemented with15 mM NaHCO_3_, 100 IU/ml penicillin, 2.5 µg/ml amphotericin B, 20 mM Na Hepes, pH 7.4, 10 µg/ml transferrin and 4 ng/ml hydrocortisone.

In order to characterize the germ cell types present in the suspension, the preparation was evaluated by flow cytometry to measure the DNA content as previously described [14]. Cells were resuspended in DMEM-F12 supplemented with 50% fetal bovine serum and fixed in ice-cold 70% ethanol. Propidium iodide was added to fixed cells to a final concentration of 50 mg/ml. Flow cytometry was performed using a FACS Caliber (Becton Dickinson). The preparation contained 27% tetraploid cells (spermatocytes) and 63% haploid cells (spermatids). Furthermore, the preparation contained small proportions of diploid cells (10%) that may represent contamination with somatic cells and spermatogonia.

### Culture conditions

Purified germ cells were plated on 10 cm^2^ dishes (5×10^6^ cells/condition) and treatment with lactate 10–20 mM or H_2_O_2_ 500 µM was performed in the absence or presence of N-acetylcysteine (1 or 5 mM), rotenone (1 µM), apocynin (500 µM), allopurinol (100 µM), α-cyano-4-hydroxy-cinnamate (10 mM) or oxamate (10 mM). Germ cells incubated for 15, 30 or 60 minutes were used for Thiobarbituric Acid Reactive Substances assay or Western blot analysis. Germ cells incubated for 24 hours were used for Northern blot analysis.

### Thiobarbituric Acid Reactive Substances (TBARS) assay

Phospholipid oxidation was determined by the colorimetric assay of TBARS [15]. Treated germ cells (5×10^6^ cells) collected by centrifugation at 400×g for 5 minutes at 4°C were resuspended with 80 µl PBS containing 0.4% w/v butylated hydroxytoluene on ice and then disrupted by ultrasonic irradiation. An aliquot (25 µl) of total cell extract (corresponding to 450 µg protein) was added to 175 µl mixed reaction solution (0.15% w/v SDS, 0.5 N HCl, 0.75% w/v phosphotungstic acid and 0.175% w/v 2-thiobarbituric acid). The mixture was heated in a boiling water bath for 45 minutes. TBARS were extracted with 200 µl of *n*-butanol. After a centrifugation at 10000×g for 5 minutes at 4°C, the absorbance at 532 nm of the butanolic phase was measured. A calibration curve was performed using malondialdehyde (MDA), generated from 1,1,3,3-tetramethoxypropane (0.4–8 µM), as standard to express the absorbance changes as nmol MDA/µg protein.

### Reactive oxygen species (ROS) assay

Germ cells (2×10^6^/condition) were loaded with the dye 2′,7′-dichlorodihydrofluorescin diacetate (H_2_DCFDA; Sigma-Aldrich) (10 µM, 15 minutes) and then treated for 30 minutes with lactate 10 mM in the absence or presence of N-acetylcysteine (5 mM)or for 15 minutes with H_2_O_2_ 500 µM in phenol red-free Minimum Essential Media without pyruvate and L-alanine. After incubation, cells were centrifuged at 400×g for 5 minutes, resuspended in 75 µl phenol red-free Minimum Essential Media without pyruvate and L-alanine, placed on a glass slide and visualized and photographed under a fluorescent light microscope (Axiophot; Carl Zeiss, Jena, Germany) [16], [17]. The percentage H_2_DCFDA-positive germ cells was calculated as (H_2_DCFDA-positive cells/total germ cells)×100.

### Western blot analysis

Treated germ cells were collected by centrifugation at 400×g for 5 minutes at 4°C and resuspended in 200 µl lysis buffer (Tris pH 7.5, 150 mM NaCl, 1 mM EDTA, 1 mM EGTA, 1% w/v Triton X100, 100 mM NaF, 10 mM Na_4_P_2_O_7_, 10 mM Na_3_VO_4_ and complete Mini, EDTA-free protease inhibitor cocktail tablets (Roche Diagnostics, Mannheim, Germany)) and incubated for 30 minutes on ice. Cell extracts were obtained by centrifugation at 16000×g for 30 minutes at 4°C. An appropriate volume of 4× Laemmli buffer (8% w/v SDS, 40% v/v glycerol, 20% v/v 2-mercaptoethanol, 0.008% w/v bromophenol blue and 250 mM Tris-HCl, pH 6.8) was added and thoroughly mixed. Samples were immersed in a boiling water bath for 5 minutes and then immediately settled on ice. Proteins (150 µg per lane seeded) were resolved in 10% SDS-PAGE (10% acrylamide/bisacrylamide for the resolving gel and 4.3% acrylamide/bisacrylamide for the stacking gel) in a Mini Protean 3 Cell (Bio-Rad, Hercules, CA, USA). After SDS-PAGE, gels were equilibrated in transfer buffer (25 mM Tris pH 8.3, 192 mM glycine, 20% v/v methanol) for 10 minutes and electrotransferred at 100 V for 60 minutes onto polyvinylidene difluoride membranes (Hybond-P, Amersham Pharmacia Biotech, Bucks, UK) using a Mini Trans-Blot Cell (Bio-Rad). Membranes were probed with commercial antibodies (Phospho-Akt (Ser473) Antibody, Akt Antibody, Phospho-p38-MAPK (Thr180/Tyr182) Antibody, p38-MAPK Antibody, Phospho-Erk1/2 (Thr202/Tyr204) Antibody and Erk1/2 Antibody; Cell Signaling Technology, Inc., Danvers, MA, USA) that allow specific recognition of phosphorylated (P-Akt, P-p38-MAPK and P-Erk1/2) and total (Akt, p38-MAPK and Erk1/2) proteins. The intensities of autoradiographic bands were estimated by densitometric scanning using NIH Image software (Scion Corporation, Frederick, MD, USA).

### Northern blot analysis

Northern blot analysis was carried out in total RNA samples isolated from treated germ cells. Extraction was performed using TRI Reagent (Sigma-Aldrich) according to the manufacturer's recommendations. The amount of RNA was estimated by spectrophotometry at 260 nm. About 10 µg total RNA was electrophoresed on a 1% agarose-10% formaldehyde gel. After migration, RNAs were transferred to Hybond-N nylon membrane (Amersham Pharmacia Biotech, Bucks, UK) by capillary transfer with 10× SSC (10× stock solution: 1.5 M NaCl and 0.15 M sodium citrate, pH 7.4) and fixed with U.V. Stratalinker (Stratagene Cloning Systems, La Jolla, CA, USA). cDNA probes were labeled with [α-^32^P]deoxy-CTP (NEN, Perkin Elmer Life and Analytical Sciences, Boston, MA, USA) using a random-primed labeling kit (Prime-a-Gene Labeling System, Promega Corporation, Madison, USA). The cDNA probes used were the following: rat LDH C, a 141b probe previously obtained using a RT-PCR technique with specific primes (5′-ACGGTCATCCTTGTTTCTTAAC-3′ and 5′-TTCATCTGGAGCTAGGTTCTGA-3′, Accession Number: NM_017266); rat MCT2 1.5 Kb insert, HindIII-BamHI [18], and rat MCT4 1.7 Kb insert, HindIII-BamHI [19] (kindly gifted by Dr. Bröer, Canberra Australia); LDH A, a rat 3′UTR 0.4 kb insert, Pst I-Bgl II [20] (kindly gifted by Dr. Jungmann, Chicago USA) and a 18S oligonucleotide. Blots were prehybridized for 3 hours at 42°C in 50% w/v formamide, 0.75 M NaCl, 20 mM sodium phosphate (pH 7.5), 1 mM EDTA, 5× Denhart's solution, 10% w/v dextran sulfate, 0.5% w/v SDS and 100 µg/ml herring sperm DNA. Hybridization was then performed overnight at 42°C in the same hybridization buffer containing 1–4×10^6^ c.p.m./ml ^32^P-labeled probe. Membranes were washed utilizing different astringency conditions depending on the probe utilized. Membranes were exposed to Hiperfilm ECL (GE Healthcare UK Limited, Buckinghamshire, UK). The 18S signal was used to standardize mRNA contents. The intensities of autoradiographic bands were estimated by densitometric scanning using NIH Image software (Scion Corporation, Frederick, MD, USA).

### Reverse Transcription-Polymerase Chain Reaction (RT-PCR)

Colon, skeletal muscle, kidney and testicular tissue, as well as, purified Sertoli and germ cell from thirty day-old rat were utilized to isolate total RNA using TRI Reagent (Sigma-Aldrich) according to the manufacturer's recommendations. The amount of RNA was estimated by spectrophotometry at 260 nm. Reverse transcription (RT) was performed on 2 µg RNA at 42°C for 50 minutes using 200 U SuperScript II reverse transcriptase enzyme (Invitrogen, Carlsbad, CA, USA) containing 125 ng random primer and 0.5 mM dNTP Mix. The cDNAs enconding NOX1, NOX2 and NOX4 were amplified from 1 µl of the cDNA reaction mixture using specific gene primers (table 1). PCR was performed with GoTaq DNA polymerase (Promega Corporation, Madison, USA) under the following conditions: initial denaturation at 94°C for 5 minutes; 35 cycles of 94°C for 30 seconds; 55°C, 57°C and 56°C (NOX1, NOX2 and NOX4 respectively) for 30 seconds and extension at 72°C for 50 seconds followed by 10 minutes at 72°C. The PCR products were resolved by 2% agarose gel and stained with ethidium bromide.

**Table 1:** Rat-specific primers sets for RT-PCR analysis.

Gene	Primer Sequence	Product Size (bp)	Accession number
NOX1	Fwd: 5′-TCTCCAAACGTGACAGTGAT-3′	112	NM_053683.1
	Rev: 5′-GGATAAACTCCATAGCTGAAGTTAC-3′		
NOX2	Fwd: 5′-ATGGAGCTGAGCGAATTGTA-3′	116	NM_023965.1
	Rev: 5′-TGGTACTGGGCACTCCTTTA-3′		
NOX4	Fwd: 5′-GTGAACGCCCTGAACTTCT-3′	158	NM_053524.1
	Rev: 5′-GCTGTAACCATGAGGAACAATA-3′		

NOX: NAD(P)H oxidase; Fwd: forward; Rev: reverse; bp: base pairs.

### Other assays

Protein content in male germ cells lysates was determined by Lowry's assay [21].

### Statistical analysis

All experiments were run in triplicates and repeated three to four times. One-way ANOVA with Tukey-Kramer post test was performed using GraphPad InStat version 3.00 for Windows 95 (GraphPad Software, San Diego, CA, USA). Probabilities <0.05 were considered statistically significant.

## Results and Discussion

### Lactate regulates MCT2 and LDH C expression with the participation of ROS

Spermatogenesis is a complex physiological process that involves cell proliferation, meiotic division, and differentiation of postmeiotic cells into spermatozoa. Postmeiotic germ cells are unable to use glucose for their energetic metabolism and they do prefer lactate as an energy source [1], [11]. The importance of lactate for normal spermatogenesis was highlighted in a report showing that spermatogenesis in adult cryptorchid rat testis is improved by intratesticular infusion of lactate [22].

**Figure 1:**
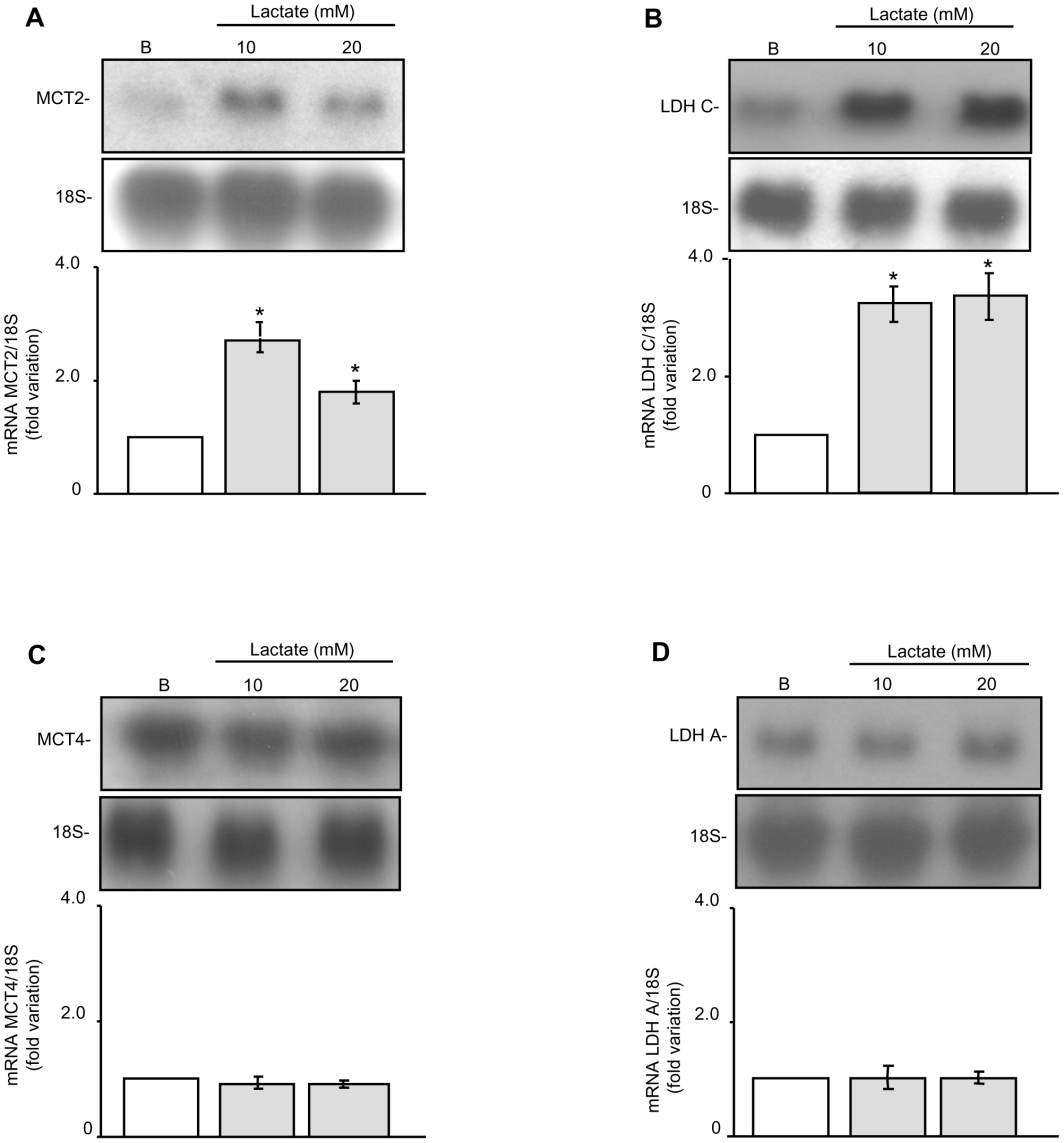
Effect of lactate on MCT2, LDH C, MCT4 and LDH A expression in germ cells. Male germ cells were incubated for 24(10 or 20 mM). Total cellular RNA was then extracted and Northern blot analysis was performed using 10 µg RNA per lane. Membranes were hybridized with labeled cDNA probes for MCT2 (**A**), LDH C (**B**), MCT4 (**C**) and LDH A (**D**). The upper panels show a representative experiment out of three. The lower panels show pooled data of three independent experiments performed indicating the fold variation in mRNA levels (ratio of mRNA to 18S in each sample) relative to basal (B). Results are expressed as means±S.D., *P<0.05 versus basal.

For much of the 20^th^ Century, lactate was largely considered a dead-end waste product of glycolysis generated under anaerobic conditions [23]. Later on, several authors showed that lactate was an important energy substrate for some cell types [1], [24]. Nowadays, various observations lead to the suggestion that, additionally, lactate might be an autocrine, paracrine or endocrine factor that modulates cell function in a distinct way [3], [25], [26]. The first objective of this investigation was to explore a possible regulation of gene expression by lactate in male germ cells. In order to achieve this goal, we analyzed whether lactate was able to regulate the expression of genes involved in its transport and metabolism. In this context, we studied the expression of the monocarboxylate transporters MCT2 and MCT4 and of the LDH subunits A and C, which are part of the enzymes catalyzing the interconversion of pyruvate and lactate. For this purpose, male germ cells were incubated for 24 hours in the absence or presence of 10 and 20 mM lactate —doses which have been shown to regulate gene expression in other cell types [3]. Figures 1A and 1B show that both lactate doses increased MCT2 and LDH C mRNA levels, respectively. Noteworthy, the same treatment did not modify MCT4 (Figure 1C) and LDH A (Figure 1D) mRNA levels. These results show that lactate effects on gene expression are rather specific. MCT2 is a monocarboxylate transporter characterized by its high affinity for lactate and by its predominant expression in those cell types, such as germ cells, which are specialized in the import of lactate [18], [27], [28], [29]. On the other hand, LDH C is the testicular isoform present only in germ cells [30], characterized by being strikingly resistant to inhibition by high lactate concentrations, thus enabling these cells to use lactate as a source of energy [31]. Noticeably, lactate did not augment the expression of MCT4, monocarboxylate transporter characterized by its low affinity for lactate and, which has been proposed to be responsible for lactate export in highly glycolytic cells [19]. LDH A, which preferentially converts pyruvate to lactate and whose expression is related to cells that produce high amounts of lactate, such as Sertoli cells, did not augment either. The results presented herein reinforce the hypothesis postulated by Hashimoto et al. [3], which points out lactate as a signalling molecule. A microarray analysis to test variations in a wide spectrum of genes involved in male germ cell function in response to lactate might bring a more complete scene of lactate acting as a signaling molecule. It is worth mentioning that lactate is not the only molecule having a dual effect as a fuel source and as a signalling molecule. This is also the case with butyrate, the main energy source of colon cells [32], which up-regulates the expression of MCT1 [33], carrier responsible for the entrance of butyrate into the cells.

**Figure 2:**
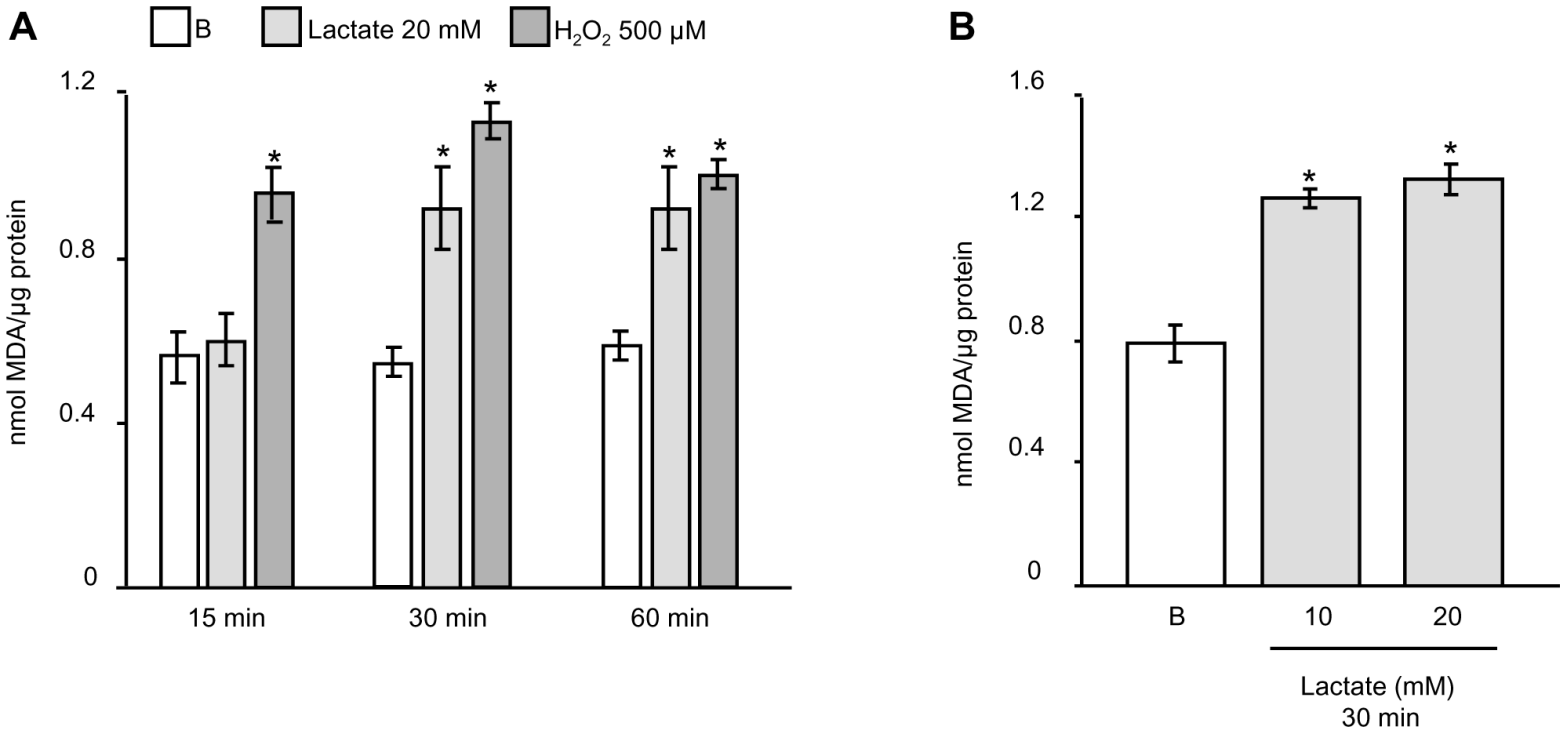
Effect of lactate on ROS production in male germ cells. Male germ cells were incubated for variable periods of time (15, 30 or 60 minutes) in the absence or presence of lactate 20 mM or H_2_O_2_ 500 µM (positive control) (**A**), or incubated for 30 minutes in the absence or presence of different doses of lactate (10 or 20 mM) (**B**). Cell extracts were prepared at the designated intervals and utilized for TBARS assay. Values are expressed as means±S.D. of triplicate incubations in one representative experiment out of three. *P<0.05 versus basal (B).

As mentioned in the introduction, the conversion of lactate to pyruvate catalyzed by LDH is accompanied by NADH production and consequently by a change in the redox balance, which may be associated with alterations in ROS levels. Therefore, we decided to examine whether lactate treatment led to a modification of ROS levels in male germ cells. Cells were incubated in the absence or presence of 20 mM lactate for variable periods of time —15, 30 and 60 minutes— or in the presence of 10 mM lactate for 30 minutes. ROS levels were indirectly estimated by the Thiobarbituric Acid Reactive Substances (TBARS) assay. Cells incubated for 15, 30 or 60 minutes in the presence of 500 µM H_2_O_2_ were utilized as a positive control. Figure 2A shows that lactate promoted a significant increase in TBARS in 30- and 60-minute incubation periods. In addition, Figure 2B shows that a lower dose of lactate —10 mM— is also able to increase TBARS in 30-minute incubation.

**Figure 3:**
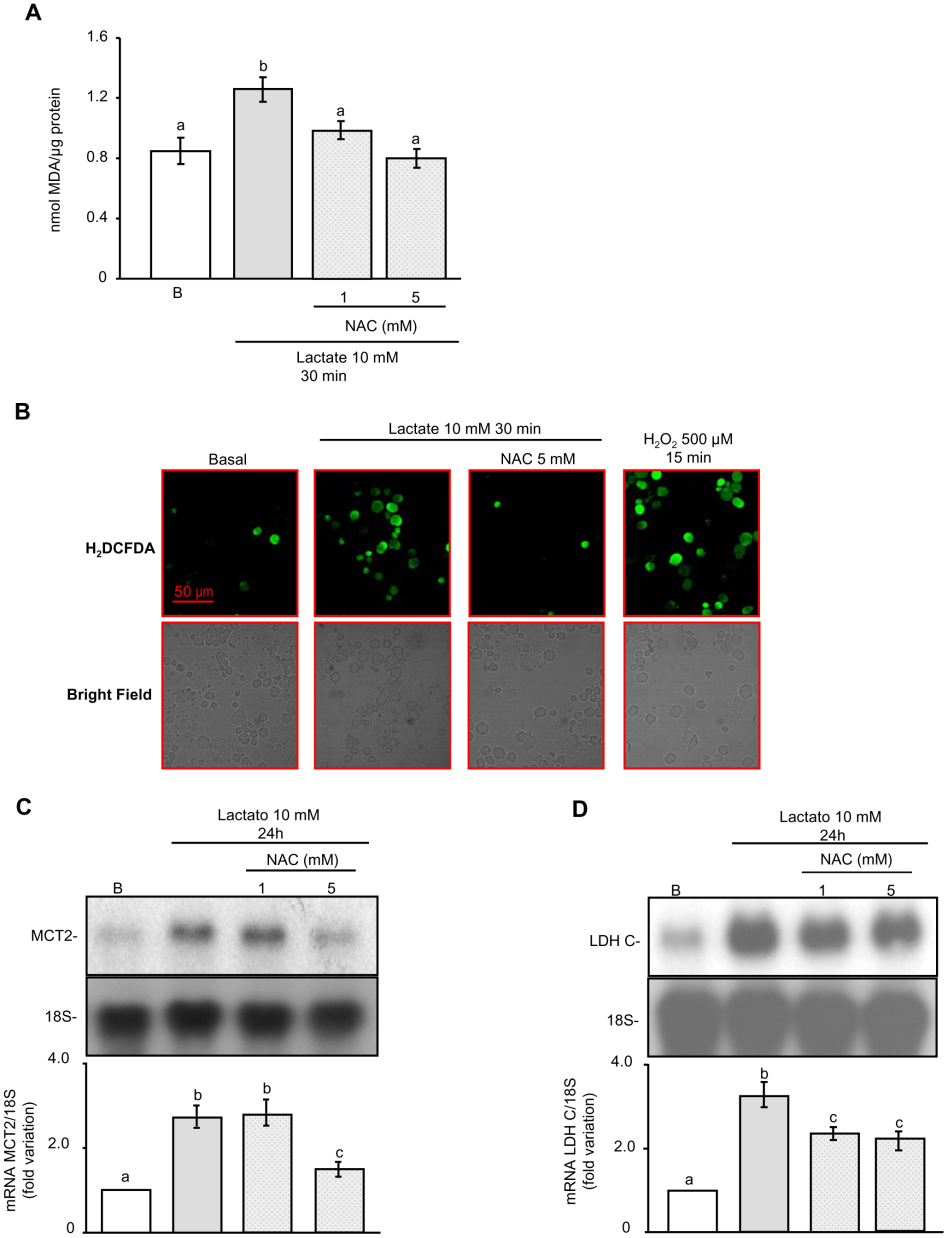
Participation of ROS in lactate actions in male germ cells. Male germ cells were incubated for 30(**A**). Values are expressed as means±S.D. of triplicate incubations in one representative experiment out of three. Different letters indicate statistically significant differences among treatment groups (p<0.05). Male germ cells pre-loaded with 10 µM 2′,7′-dichlorofluorescin diacetate (H_2_DCFDA) were incubated for 30 minutes without (Basal) or with lactate 10 mM in the absence or presence of N-acetylcysteine (5 mM). After incubation, cells were centrifuged and then placed on a glass slide for observation at ×400 magnification. A positive control was included (H_2_O_2_ 500 µM for 15 minutes) (**B**). Images are representative of three independent experiments. Scale bar: 50 µm. Male germ cells were incubated for 24 hours in the absence or presence of lactate 10 mM without or with NAC 1 or 5 mM. Total cellular RNA was then extracted and Northern blot analysis was performed using 10 µg RNA per lane. Membranes were hybridized with labeled cDNA probes for MCT2 (**C**) and LDH C (**D**). The upper panels show a representative experiment out of three. The lower panels show pooled data of three independent experiments performed indicating the fold variation in mRNA levels (ratio of mRNA to 18S in each sample) relative to basal (B). Results are expressed as means±S.D. Different letters indicate statistically significant differences among treatment groups (P<0.05).

Next, and in order to analyze whether elevated levels of ROS and regulation of gene expression produced by lactate were somehow related, we decided to combine lactate treatment with the ROS scavenger N-acetylcysteine (NAC) —1 or 5 mM. Figure 3A shows that, as expected, co-incubation of lactate with NAC decreased TBARS levels. ROS were also detected by the oxidant-sensing probe 2′,7′-dichlorodihydrofluorescin diacetate and examinated by fluorescence microscopy. The images obtained confirmed that lactate raised ROS and that NAC again decreased lactate-stimulated ROS production (Figure 3B). The percentage H_2_DCFDA-positive germ cells was calculated as (H_2_DCFDA-positive cells/total germ cells)×100 (table 2). Furthermore, we observed that NAC partially blocked the effect of lactate on the regulation of MCT2 (Figure 3C) and LDH C (Figure 3D) mRNA levels. These results suggest that ROS may act as mediators of lactate actions in male germ cells. These results are in agreement with those previously shown by Végran et al. [26] in HUVEC cells, where lactate stimulation of IL-8 expression was mediated by ROS.

**Table 2:** H_2_DCFDA positive cells quantification.

	Positive germ cells
Basal	16±8
Lactate	40±10*
Lactate+NAC	9±6
H_2_O_2_	49±12*

Each condition represents %H_2_DCFDA-positive germ cells (∼400 cells/group). Results are presented as means±S.D. of one representative experiment out of 4.

* P<0.05 vs Basal.

### ROS mediate lactate regulation of Akt- and p38-MAPK-signalling pathways

**Figure 4:**
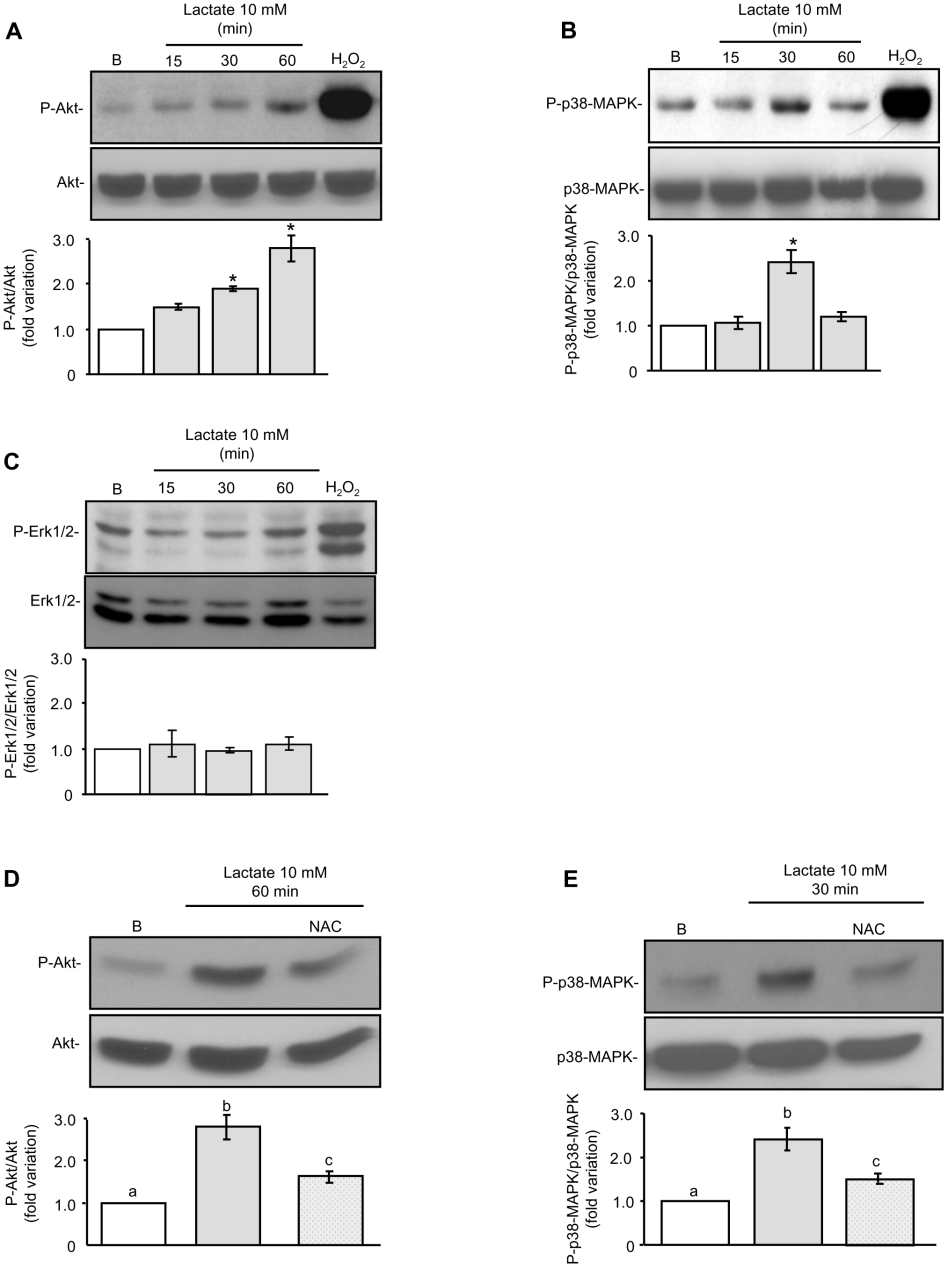
Effect of lactate on P-Akt, P-p38-MAPK and P-Erk1/2 levels in male germ cells. Male germ cells were incubated for variable periods of time (15, 30 or 60 minutes) in the absence or presence of lactate 10 mM with or without NAC 5 mM or incubated for 15 minutes with H_2_O_2_ 500 µM. Cell extracts were prepared at the designated intervals and utilized for Western blot analysis using specific antibodies for P-Akt and Akt (**A, D**), P-p38-MAPK and p38-MAPK (**B, E**) or P-Erk½ and Erk½ (**C**). The autoradiographies show a representative experiment out of three. The lower panels show pooled data of three independent experiments indicating the fold variation in phosphorylation (ratio of P-Akt, P-p38-MAPK and P-Erk1/2 to Akt, p38-MAPK and Erk1/2 respectively in each sample) relative to basal (B). Results are expressed as means ± S.D. *P<0.05 versus basal. Different letters indicate statistically significant differences among treatment groups (P<0.05).

As mentioned in the introduction, recent findings suggest that the initiation and/or proper functioning of several signal transduction pathways, such as PI3K/Akt, p38-MAPK and Erk1/2, may be involved in the mechanism of action of ROS [7], [8], [9]. In this context, the redox-dependent protein modification has been recognized as an important mechanism in signal transduction [34]. Considering these observations, we designed experiments to analyze the ability of lactate to regulate cell signaling pathways and a possible participation of ROS in this regulation. For this purpose, male germ cells were incubated in the absence or presence of 10 mM lactate for variable periods of time and the levels of phosphorylated Akt (P-Akt), p38-MAPK (P-p38-MAPK) and Erk1/2 (P-Erk1/2) were determined. Figures 4A and 4B show that lactate promoted a time-dependent increase in P-Akt and in P-p38-MAPK levels. On the other hand, Figure 4C shows that lactate did not modify P-Erk1/2 levels. These figures also show that incubation of germ cells with 500 µM H_2_O_2_ for 15 minutes increased P-Akt, P-p38-MAPK and P-Erk1/2 levels.

In order to analyze a possible role of ROS in lactate regulation of P- Akt and P-p38-MAPK levels we combined lactate treatment with NAC. Figure 4D and 4E show that NAC partially inhibited the ability of lactate to raise P-Akt and P-p38-MAPK levels. The fact that H_2_O_2_ but not lactate activated Erk1/2 may be related to different levels of ROS attained by both treatments or alternatively to the specificity of the response. Even though H_2_O_2_ is widely used as an experimental exogenous ROS, it has to be kept in mind that treatment of a cell with H_2_O_2_ may not adequately reflect endogenous ROS signaling. For instance, exogenous H_2_O_2_ produces broad signaling responses in endothelial cells, which include the activation of Erk1/2, JNK and p38-MAPKs. In contrast, the EGF-stimulated signaling response in the above-mentioned cell type, which is known to be mediated by ROS, is restricted to the mitogenic Erk1/2 pathway [35].

### Possible mechanisms involved in lactate regulation of ROS levels

Evidence obtained so far allows us to conclude that lactate acting on germ cells is able to regulate ROS levels, signal transduction pathways and gene expression and that these phenomena may be, at least in part, related. The mechanism of action of lactate that elicits biological responses in different cell types is mostly unknown. Particularly, no information is available on the molecular mechanisms that may be involved in lactate actions in germ cells beyond its energetic function.

**Figure 5:**
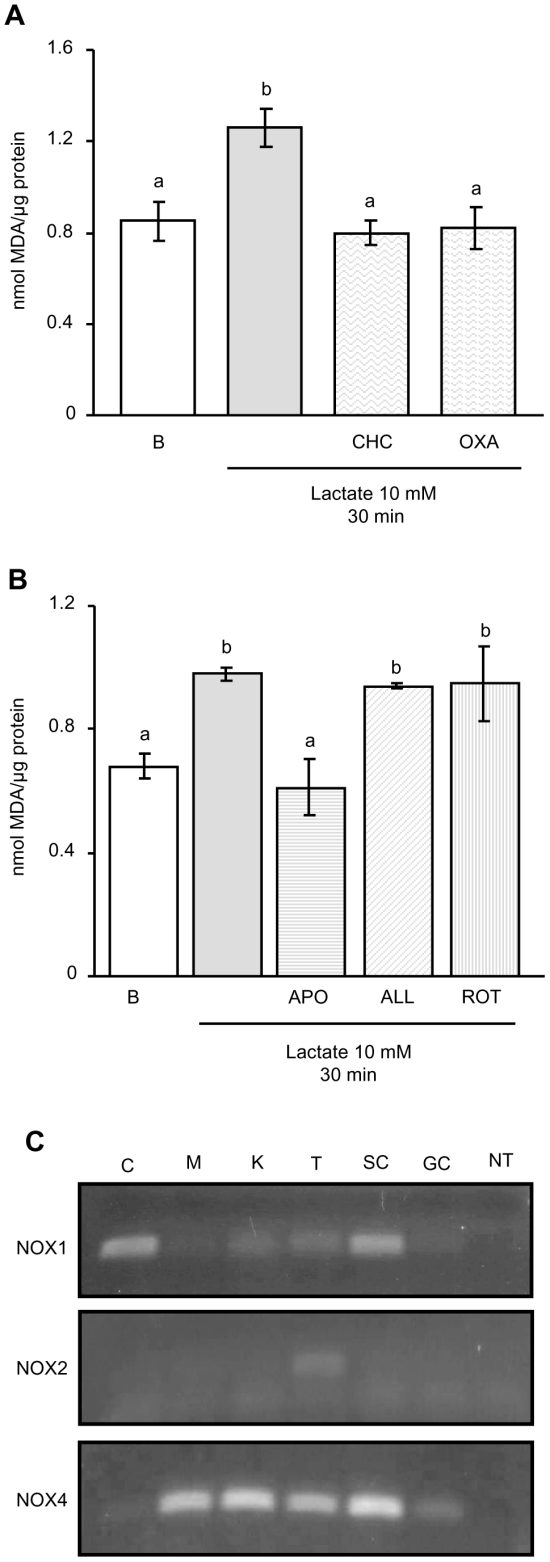
(A) and (B) Mechanisms involved in lactate regulation of ROS levels in male germ cells. Male germ cells were incubated for 30α-cyano-4-hydroxy-cinnamate (CHC) 10 mM, oxamate (OXA) 10 mM (**A**), apocynin (APO) 500 µM, allopurinol (ALL) 100 µM or rotenone (ROT) 1 µM (**B**). Cell extracts were prepared and utilized for TBARS assay. Values are expressed as means±S.D. of triplicate incubations in one representative experiment out of three. Different letters indicate statistically significant differences among treatment groups (p<0.05). (**C**) *Expression of NOX family members in male germ cells.* Total RNA of rat colon (C), skeletal muscle (M), kidney (K), testis (T), Sertoli cells isolated as previously described [44] (SC) and male germ cells (GC) were extracted and analyzed by RT-PCR and visualized by ethidium bromide staining. NT is no template control.

It is relevant to mention here that a G-protein-coupled receptor, GPR81, with the ability to bind lactate has been described [36]. In addition, it has been observed that lactate exerts antilipolytic effects in adipocytes through interaction with this receptor [25], [36]. We hypothesized that the observed effects of lactate in germ cells rather than being associated with binding to GPR81 might be associated with its oxidation to pyruvate after entering into the cell. In order to evaluate this hypothesis, we decided to impair: a) its entrance into the cell by means of inhibiting MCT transporters with α-cyano-4-hydroxy-cinnamate (CHC) and b) its oxidation to pyruvate by means of inhibiting LDH activity with oxamate (OXA). Male germ cells were incubated for 30 minutes with lactate 10 mM in the absence or presence of either CHC 10 mM or OXA 10 mM. Figure 5A shows that in the presence of either CHC or OXA, lactate was unable to increase ROS production. Given the fact that inhibition of lactate transporters blocked lactate effects on ROS production, it is tempting to speculate that the actions of lactate in male germ cells are not related to actions through GPR81 but to its entrance into the cells.

As for the sources of ROS, it is well known that oxidation of lactate is accompanied by NADH generation, that NADH may constitute a substrate of NADH/NADPH oxidases (NOXs) and that NOXs activities generate ROS. Other sources of ROS in a cell, such as activities of xanthine oxidase and NADH dehydrogenase, should be considered. To evaluate which of these different sources of ROS might be participating in lactate effects, male germ cells were incubated for 30 minutes with 10 mM lactate in the absence or presence of either 500 µM apocynin (APO) —inhibitor of NOXs; 100 µM allopurinol (ALL) — inhibitor of xanthine oxidase; or 1 µM rotenone (ROT) —inhibitor of NADH dehydrogenase. Figure 5B shows that only apocynin, the NOX inhibitor, was able to impair the effect of lactate on ROS production. The obtained evidence is consistent with a role of NOX activity in lactate effect. The participation of NOX in the mechanism of action of lactate to produce ROS has been previously suggested. In this respect, Oeckler et al. [37] have proposed that lactate can substantially modulate endothelium bovine pulmonary arteries NOX activity and hence ROS production, via a mechanism involving the elevation of cytosolic NADH. Considering these previous observations, increased ROS production in male germ cells by lactate may be interpreted as the consequence of elevated levels of NADH, substrate of NOX, resulting from oxidation of lactate. This hypothesis gains support by the fact that OXA, an inhibitor of LDH, blocks lactate effects on ROS production.

As for NOX, these enzymes have been shown to be involved in the regulation of a wide range of physiological functions, including cell death and survival, differentiation, proliferation, Ca^2+^ signalling, gene expression and migration [38], [39]. It has been observed that NOX family members are variably expressed in different cell types [40]. In the rat, four members have been identified —NOX1-4. NOX3 is only expressed in the inner ear [41]. Consequently, we decided to analyze the expression of NOX1, NOX2 and NOX4 in male germ cell. Figure 5C shows that male germ cells only express NOX4. Unlike other NOX proteins, NOX4 can generate ROS in the absence of exogenous stimuli and it has been proposed that its activity may be regulated by the local availability of the substrate NAD(P)H [42], [43].

**Figure 6:**
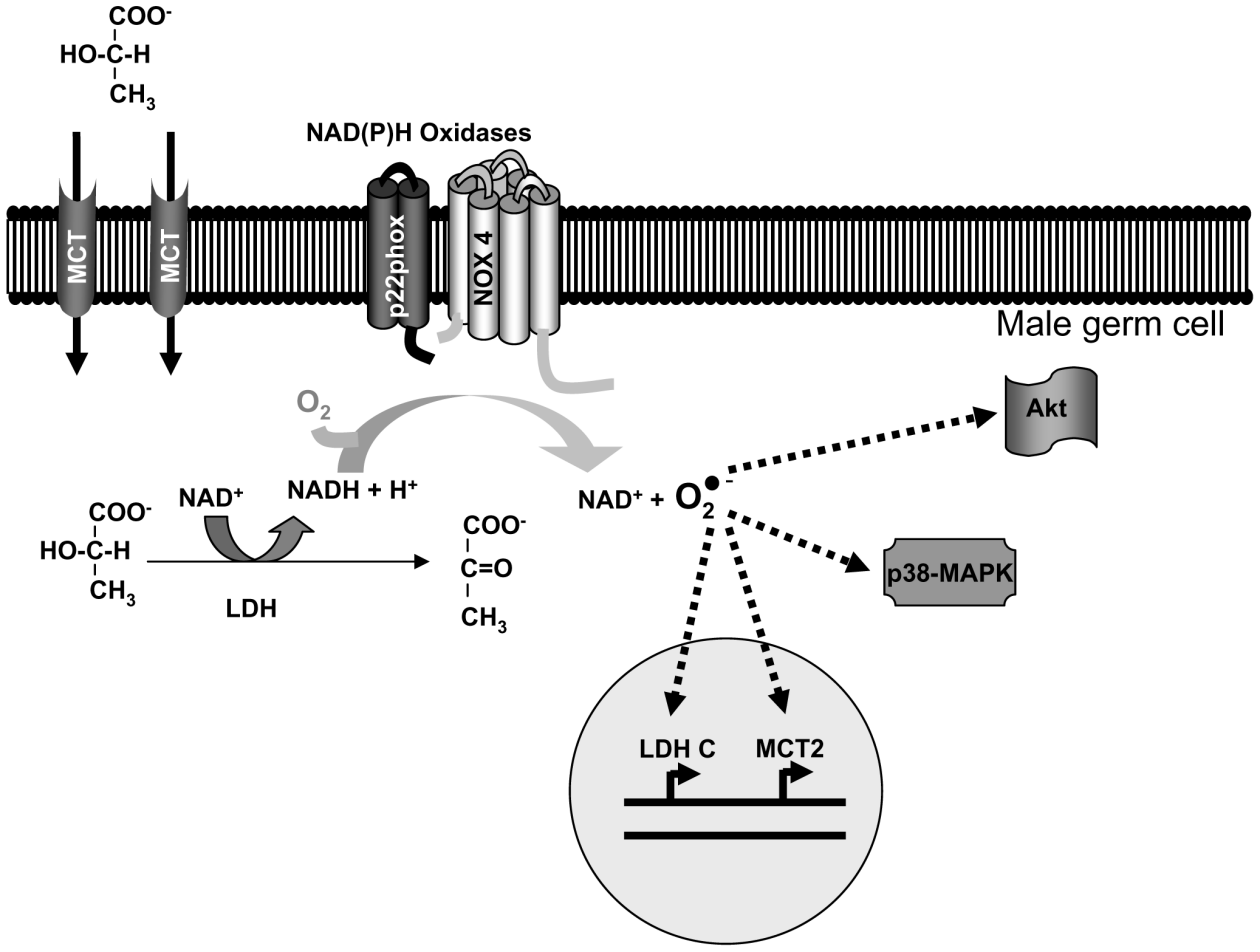
Proposed mechanism of lactate effects on male germ cell function. See “Discussion” for details.

In summary, our results are consistent with a scenario where lactate, taken up by germ cells, becomes oxidized to pyruvate with the resultant increase in NADH, which is a substrate for NOX4. ROS, products of NOX4 activity, may act as second messengers regulating signal transduction pathways and gene expression (Figure 6).

Our study provides novel evidence for a role of lactate as a signalling molecule in the seminiferous tubule. In this regard, lactate might be considered a paracrine factor secreted by Sertoli cells that, in addition to being a source of energy, regulates germ cell functioning.
